# Bioinformatics and Expression Analyses of the *TaATLa* Gene Subfamily in Wheat (*Triticum aestivum* L.)

**DOI:** 10.3390/ijms252212454

**Published:** 2024-11-20

**Authors:** Yifei Chen, Kexin Zhao, Heng Chen, Luzhen Wang, Shuai Yan, Lei Guo, Jianjun Liu, Haosheng Li, Danping Li, Wenjia Zhang, Xiaoyan Duan, Xiukun Liu, Xinyou Cao, Xin Gao

**Affiliations:** 1 College of Agronomy, Northwest A&F University, Yangling 712100, China; chenyifei@nwafu.edu.cn (Y.C.);; 2 Crop Research Institute, Shandong Academy of Agricultural Sciences/National Engineering Research Center of Wheat and Maize/State Key Laboratory of Wheat Improvement/Key Laboratory of Wheat Biology and Genetic Improvement in North Yellow & Huai River Valley/Shandong Provincial Technology Innovation Center for Wheat, Jinan 250100, China13121259599@163.com (D.L.);; 3 Shandong Academy of Agricultural Sciences, Jinan 250100, China

**Keywords:** wheat (*Triticum aestivum* L.), amino acid transporter, TaATLa subfamily, bioinformatics analysis, gene expression

## Abstract

Amino acids are the main form of nitrogen in plants, and their transport across cell membranes relies on amino acid transporters (AATs). Among the plant AATs, the TaATLa subfamily comprises 18 members, yet the bioinformatics characteristics and functions of *TaATLa* genes in common wheat remain poorly understood due to their complex genomes. This study performed genomic analyses of *TaATLas*. These analyses included chromosome distributions, evolutionary relationships, collinearity, gene structures, and expression patterns. An analysis of cis-acting elements and predicted miRNA-*TaATLas* regulatory networks suggests that *TaATLas* are regulated by light, hormones, and stress signals. Functional assays revealed that TaATLa6 transports glutamine (Gln), glutamate (Glu), and aspartate (Asp) in yeast. In contrast, TaATLa4 specifically transports Gln and Asp. Furthermore, *TaATLas* exhibits diverse gene expression patterns, with *TaATLa4-7D* enhancing yeast heat tolerance in a heterologous expression system, indicating its potential role in adapting to environmental stress by regulating amino acid transport and distribution. This study sheds light on the functional roles of *TaATLa* genes, with implications for improving nitrogen use in wheat and other crop species.

## 1. Introduction

Amino acids are essential for plant growth and development, serving as building blocks for enzymes and proteins [1]. Amino acid transporters (AATs), which are integral membrane proteins encoded by multiple gene families, have been extensively studied in yeast, mammals, and plants [2]. AATs play a critical role in plant metabolism, regulating amino acid mobilization and transport, which are crucial for development and yield formation [2,3,4]. To understand the functions of *AAT* genes in plants, genome-wide identification and annotation are essential. AATs in plants are categorized into two main families based on sequence similarity and transport properties: the amino acid/auxin permease (AAAP) family, also known as the amino acid transporter family (ATF), and the amino acid-polyamine-choline (APC) transporter family [5,6]. The AAAP family includes several subfamilies, such as amino acid permeases (AAPs), amino acid transporter-like a/b (ATLa/b), lysine and histidine transporters (LHTs), γ-aminobutyric acid transporters (GATs), neutral amino acid transporters (ANTs), auxin transporters (AUXs), and proline transporters (ProTs), while the APC family comprises three subfamilies: cationic amino acid transporters (CATs), amino acid/choline transporters (ACTs), and polyamine H^+^-symporters (PHSs) [7,8,9].

Advancements in sequencing technologies have led to the characterization of *AATs* across various plant species [2]. For instance, 85, 94, 72, 189, and 296 *AAT* genes have been identified in rice (*Oryza sativa* L.), millet (*Setaria italica* L.), potato (*Solanum tuberosum* L.), soybean (*Glycine max* L.), and wheat (*Triticum aestivum* L.), respectively [4,7,10,11,12]. In recent studies, the *Ataap2* mutant exhibits increased nitrogen allocation to leaves under different nitrogen conditions, thereby improving their photosynthetic capacity [13]. A CRISPR mutant of *OsAAP12* enhanced rice tillering and grain yield through the regulation of amino acid and cytokinin levels [14]. In tea trees, CsAAP7.2 facilitated the absorption of amino acids from the soil and transported them over long distances [15]. Evolutionarily related genes within the same subfamily often show distinct localization and functional roles [16,17,18]. For instance, *OsAAP6* overexpression increases amino acid uptake in roots and enhances storage protein expression [17], while silencing *OsAAP3* expression reduces the levels of aspartate (Asp), threonine (Thr), serine (Ser), isoleucine (Ile), leucine (Leu), lysine (Lys), and arginine (Arg) in straw but unexpectedly enhances tiller number and grain yield [18]. Therefore, it is necessary to identify and experimentally validate the functions of each member within the *AAT* gene family.

Wheat is a major cereal crop globally, vital for food security. In 2022, global wheat production reached roughly 808.44 million tons, according to the Food and Agriculture Organization of the United Nations (FAO) (https://www.fao.org/faostat/en/#home, accessed on 22 September 2024). Nitrogen is a key factor in wheat production [19], but excessive N fertilizer use increases production costs and has negative environmental impacts [20]. Wheat’s nitrogen use efficiency (NUE) is relatively low compared to other staple crops, with only about one-third of applied nitrogen being absorbed [21]. Therefore, comprehensively understanding the molecular mechanisms of NUE is crucial for developing wheat varieties with improved efficiency [22].

Abiotic and biotic stresses, including heat, cold, drought, and pathogens, also affect wheat yield and quality. Wheat has evolved complex regulatory mechanisms to cope with these stresses [23]. In rice, certain *AATs* (e.g., *OsAAP15*, *OsATL6*, and *OsANT3*) were upregulated under drought, salt, and cold conditions, while others, including *OsAUX1*, *OsAAP4*, *OsBAT4*, and *OsAAP8*, were downregulated [4]. In wheat, *TaAAP2* expression in leaves decreased sharply under drought and fluctuated with prolonged heat stress [12]. Thus, investigating the expression of *AAT* genes in response to stress may provide insights for breeding wheat varieties with enhanced stress resilience.

A genome-wide analysis in bread wheat identified 296 high-confidence *AAT* genes, including 204 in the AAAP family and 92 in the APC family, showing diverse expression across tissues and developmental stages [12]. However, the complexity of wheat genome, comprising three homologous subgenomes (A, B, and D) and exceeding 17 Gb, has hindered the detailed study of the *ATLa* gene subfamily in wheat.

This study conducted a comprehensive genome-wide investigation of the *TaATLa* gene subfamily utilizing wheat genome and protein databases. Phylogenetic relationships, chromosomal locations, collinearity across species, gene structures, cis-elements in promoters, and miRNA targets for the 18 identified *TaATLa* genes were systematically analyzed. Expression patterns were evaluated across tissues and stress conditions, with two *TaATLa* genes subjected to functional analysis under heat stress using a yeast heterologous expression system. The amino acid transport functions of TaATLas were determined using yeast complementation. Our findings provide insights into the evolutionary history and functional roles of *TaATLa* genes, laying the groundwork for future research on *AATs* in wheat and other crops.

## 2. Results

### 2.1. Genome-Wide Identification and Analysis of TaATLa Genes

We conducted a comprehensive genome-wide analysis to identify members of the *TaATLa* subfamily in *Triticum aestivum* utilizing sequence data from five *Arabidopsis* and six rice *ATLa* genes through BLASTp searches. This analysis revealed 18 *TaATLa* genes in wheat, which were designated based on their chromosomal locations (Table 1). These genes exhibit diverse gene structures, with coding sequence (CDS) lengths ranging from 1461 bp (*TaATLa6-7A*) to 7247 bp (*TaATLa3-6A*), and typically contain four introns, except for *TaATLa6*. The *TaATLa* genes encode polypeptides with 445 (*TaATLa3-6B*, *-6D*) to 491 (*TaATLa6-7B*, *-7D*) amino acid residues, molecular weights (MW) of 47.521 kD (*TaATLa3-6D*) to 52.199 kD (*TaATLa6-7B*), and predicted isoelectric points (PI) of 5.92 (*TaATLa6-7A*) to 9.27 (*TaATLa1-3A*, *-3B*). These *TaATLa* genes contain 10 to 12 transmembrane (TM) regions. Subcellular localization predictions indicate that all TaATLa proteins are located in the plasma membrane system.

### 2.2. Phylogenetic Analysis of TaATLa Proteins

**Figure 1:**
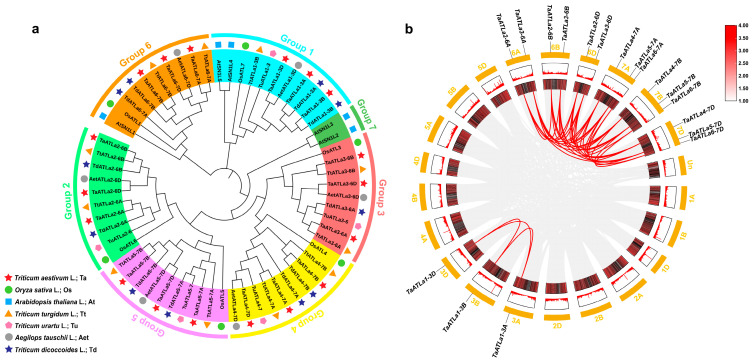
Evolutionary analysis of TaATLas. (**a**) Phylogenetic tree of ATLa proteins is constructed by the neighbor-joining method using MEGA11 from the following species: Ta, *Triticum aestivum* L. (18); At, *Arabidopsis thaliana* L. (5); Os, *Oryza sativa* L. (6); Td, *Triticum dicoccoides* L. (11); Tu, *Triticum urartu* L. (5); Aet, *Aegilops tauschii* L. (6); and Tt, *Triticum turgidum* L. (11). Based on the homologous genes of ATLa in wheat, 62 proteins are divided into 7 groups and marked with different colors. (**b**) Distribution and duplication events of *TaATLa* genes across the wheat genome. All typical *TaATLa* genes are mapped to 21 wheat chromosomes in a circle using Circos tool, and segmental duplications are mapped to their respective locations. Gray regions indicate all synteny blocks within the wheat genome, while red lines represent segmental duplications. The chromosome numbers are marked outside of the circle.

To elucidate the phylogenetic relationships of ATLa proteins across species and discern evolutionary patterns in wheat and its progenitors, we constructed a phylogenetic tree (Figure 1a and Table S1) based on bootstrap values. The overall phylogenetic structure classified the 62 ATLa proteins into seven distinct groups. Groups 1–6 each contains three homoeologous members in wheat, while Group 7 consists of AtSN1L2 and AtSN1L3. The TaATLa proteins demonstrate close evolutionary relationships with their progenitor species (TdATLas, TuATLas, AetATLas, and TtATLas) rather than with their wheat homoeologous copies. This observation suggests that *ATLa* genes likely diverged from a common ancestor with minimal genetic changes during the evolution of wheat and its ancestors (Figure 1a).

### 2.3. Chromosome Localization and Collinearity Assessment of the TaATLa Gene Subfamily

Wheat, as a hexaploidy plant, possesses subgenomes (A, B, and D), meaning each gene may have orthologues on three homologous chromosomes [24]. Chromosome localization analysis revealed that *TaATLa* genes are distributed across nine chromosomes (Figure S1). The three *TaATLa1* genes on chromosome 3 are evenly spread across subgenomes A, B, and D. Chromosomes 6A, 6B, and 6D each contains two *TaATLa* genes, while chromosomes 7A, 7B, and 7D each hosts three *TaATLa* members. To explore the role of segmental duplication in the expansion of plant *TaATLa* gene family, a one-step MCScanX analysis was performed [25]. This analysis revealed 36 duplication events: 18 segmental duplication events between homologous chromosomes and 18 between non-homologous chromosomes (Figure 1b). No tandem duplication was observed, suggesting that segmental duplication is the primary driver of *TaATLa* gene expansion. The ratio of non-synonymous substitutions (Ka) to synonymous substitutions (Ks) was calculated for these 36 paralogous gene pairs to assess the selection pressure on the protein-coding genes (Table S2). A Ka/Ks ratio greater than 1 (Ka/Ks > 1) suggests a positive selection and adaptive evolution, while a Ka/Ks ratio of 1 (Ka/Ks = 1) indicates neutral evolution without selective pressure. Conversely, a Ka/Ks ratio less than 1 (Ka/Ks < 1) implies purifying or negative selection, which removes deleterious mutations and conserves gene function [26]. All pairs had a Ka/Ks ratio below 1.0, ranging from 0.021 to 0.153, indicating a strong purifying selection, which helps maintain the functional stability of *TaATLa* genes during evolution.

**Figure 2:**
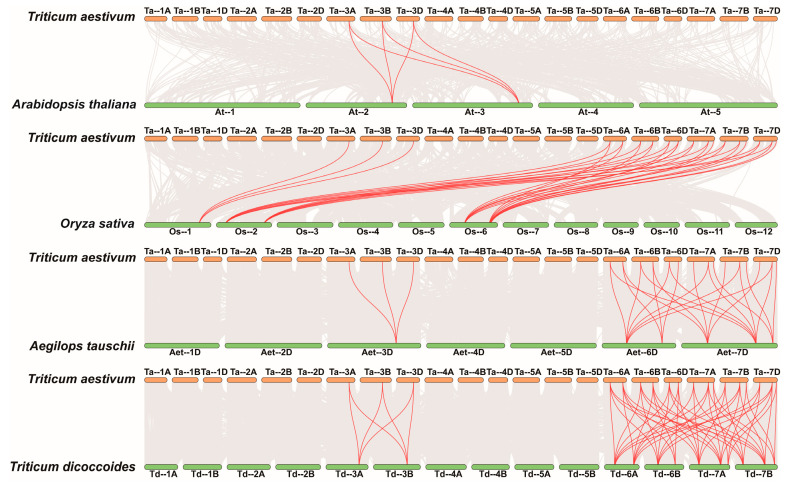
Collinearity analysis of *ATLa* genes by individually comparing *Triticum aestivum* with *Arabidopsis thaliana*, *Oryza sativa*, *Aegilops tauschii*, and *Triticum dicoccoides*. Gray lines in the background represent the collinear blocks of the plant genome and red lines in highlight indicate the syntenic *ATLa* gene pairs.

To gain a more comprehensive understanding of the evolutionary trajectory of the *ATLa* gene subfamily, synteny analysis was conducted by pairwise comparison of *Triticum aestivum* (AABBDD, hexaploid) with four species: *Arabidopsis thaliana* (diploid), *Oryza sativa* (diploid), *Aegilops tauschii* (DD, diploid), and *Triticum dicoccoides* (AABB, tetraploid) (Figure 2). The analysis revealed 6, 30, 26, and 56 syntenic genes between *Triticum aestivum* and the respective species. These findings suggest a shared genetic lineage and indicate the potential presence of homologous gene pairs that predate the divergence of their ancestral plant species.

### 2.4. Structures and Conserved Motifs Analysis of TaATLa Genes

**Figure 3:**
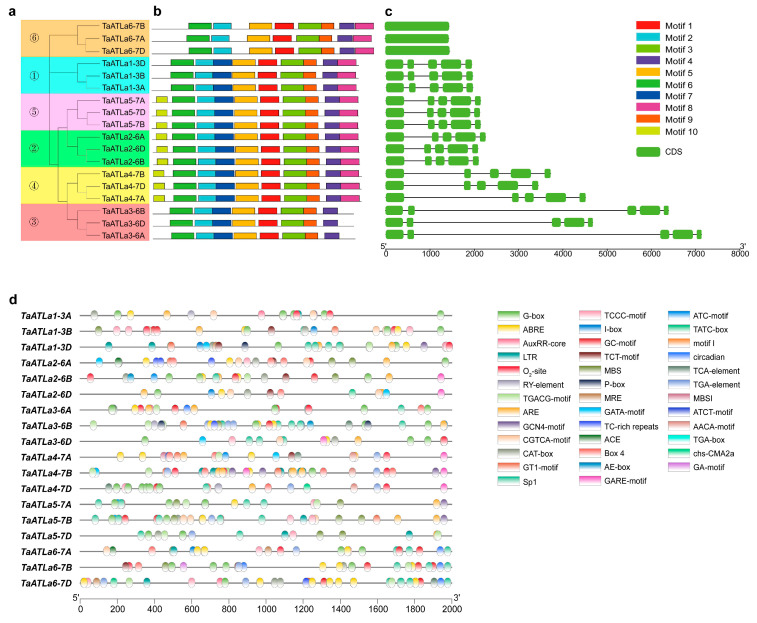
Gene structures and conserved motifs of *TaATLa* genes, and the prediction of cis-acting elements of *TaATLa* promoters. (**a**) The neighbor-joining (NJ) phylogenetic tree was constructed with protein sequences encoded by the longest transcript of *TaATLa* genes with bootstrap values of 1000 replicates. (**b**) Distribution of all motifs identified by MEME. Differently coloured frames represent different protein motifs. (**c**) Gene structures of the 18 *TaATLa* genes. The green rectangles in gene structures represent the coding sequences (CDSs), and the black lines represent introns. (**d**) Predicted cis-acting elements of *TaATLa* promoters by PlantCARE. The different cis-acting elements are represented by differently coloured boxes. Names of cis-acting elements are shown on the right.

To gain insights into the function of *TaATLa* genes, we analyzed their conserved protein motifs, identifying 10 distinct motifs (Figure 3a,b and Figure S2). All TaATLa proteins share motifs 1–6, and 9. However, TaATLa1 lacks motif 10, TaATLa3 is missing motifs 8 and 10, and TaATLa6 lacks motifs 7 and 10. Orthologous gene clusters exhibit similar motif profiles, suggesting minimal functional divergence among these genes [27]. The exon–intron structure provides crucial information about gene evaluation and functional diversification [28]. Using TBtools (v2.061) Gene Structure View, we performed a detailed analysis of *TaATLa* genes’ exon–intron structure (Figure 3c). *TaATLa6* contains only one exon, while the other members possess four introns and five exons each. Notably, *TaATLa3-6A* has the longest length, and *TaATLa6-7A* is the shortest.

### 2.5. Cis-Acting Elements in the Promoter Regions of TaATLa Genes

Cis-acting elements in gene promoters play a key role in regulating gene expression [29]. To elucidate the transcriptional regulation mechanism and potential gene function, we retrieved 2000 bp sequences upstream of the transcription start site of each *TaATLa* gene from the Ensembl Plants Database (https://plants.ensembl.org/Triticum_aestivum/Info/Index, accessed on 14 February 2024) and analyzed them using PlantCARE (https://bioinformatics.psb.ugent.be/webtools/plantcare/html/, accessed on 14 February 2024) (Figure 3d). The identified cis-elements were categorized into four groups: light response, hormone response, stress response, and growth and development (Table S3). The analysis revealed 15 light-responsive elements, such as ATC-motif, GATA-motif, and TCCC-motif, suggesting that *TaATLa* genes are influenced by light. Ten hormone-responsive elements were also identified, including ABRE (responsive to abscisic acid), AuxRR-core, TGA-box, and TGA-element (responsive to auxin), CGTCA-motif, and TGACG-motif (responsive to MeJA), GARE-motif, P-box, TATC-box (responsive to gibberellin), and TCA-element (responsive to salicylic acid). Additionally, elements involved in stress responses like low-temperature, drought, and hypoxia responsiveness were found, alongside motifs related to growth and development, such as AACA-motif, GCN4-motif, CAT-box, motif I, RY-element, circadian, O_2_-site, and MBSI. These findings indicate that *TaATLa* genes likely contribute to wheat growth and development and responses to environmental stresses.

### 2.6. Analysis of MicroRNAs Targeting TaATLa Genes

**Figure 4:**
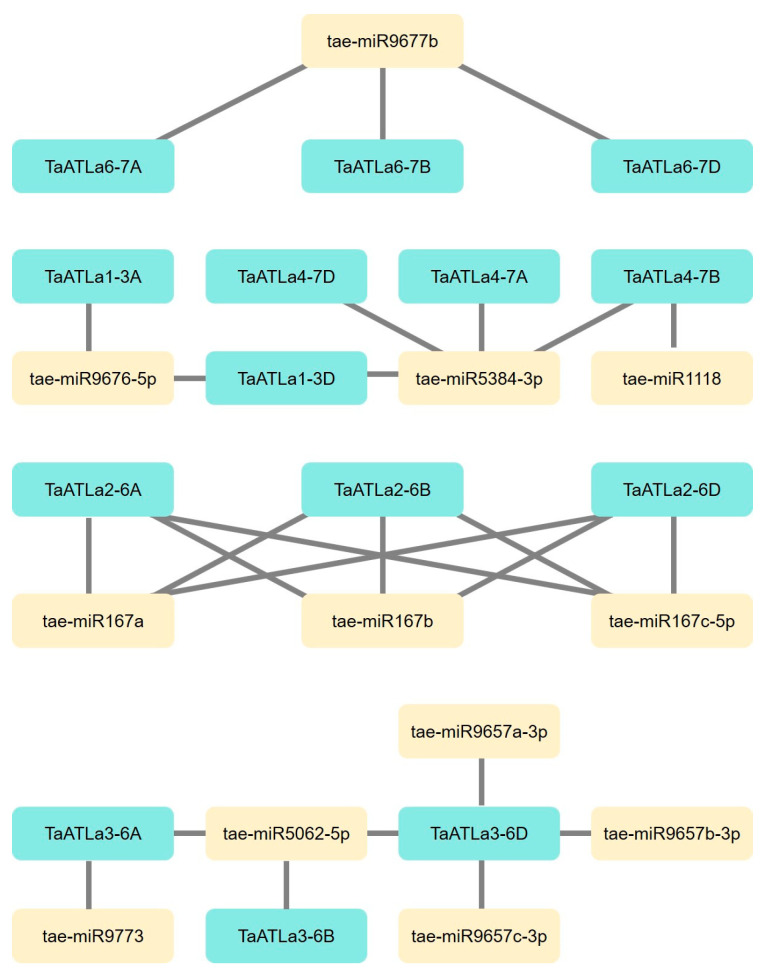
A representation of the regulatory network between the putative miRNAs and their targeted *TaATLa* genes. Blue boxes represent *TaATLa* genes and beige boxes represent targeted miRNAs.

MicroRNAs (miRNAs) are small non-coding RNAs that regulate gene expression by recognizing specific sequences and interfering with transcriptional, translational, or epigenetic mechanisms in both animals and plants [30]. To elucidate the potential regulatory role of miRNAs on *TaATLa* genes, we used psRNATarget tools (https://www.zhaolab.org/psRNATarget/, accessed on 22 September 2024) [31] for miRNA-target prediction. The analysis revealed 12 distinct miRNAs targeting 14 *TaATLa* genes (Figure 4). Some miRNAs regulate multiple *TaATLa* members, such as tae-miR9677b, which targets three *TaATLa6* members, and tae-miR167a, tae-miR167b, and tae-miR167c-5p, which concurrently target three *TaATLa2* members. Additionally, tae-miR9676-5p targets both *TaATLa1-3A* and *TaATLa1-3D*, and tae-miR5384-3p regulates four *TaATLa* members. Certain miRNAs, including tae-miR1118, tae-miR9773, and tae-miR9657a-c, specifically target only one *TaATLa* gene. These regulatory networks may provide insights into the functional roles of *TaATLa* genes and serve as a foundation for future research.

### 2.7. Expression Profile of TaATLa Genes

**Figure 5:**
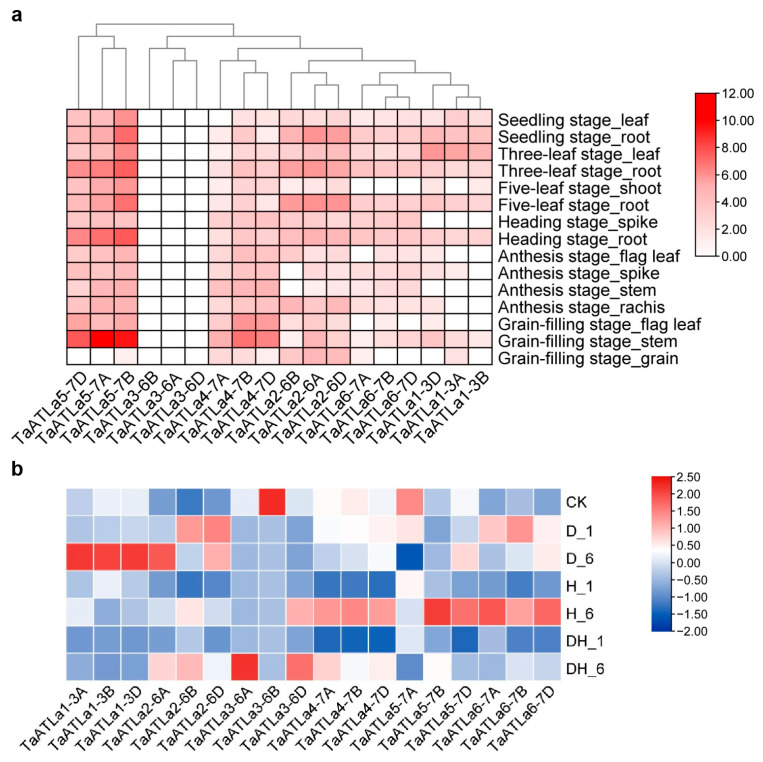
Expression pattern analysis of *TaATLa* gene subfamily. (**a**) Heatmap of *TaATLas* expression in a variety of tissues at different stages. (**b**) Heatmap of *TaATLas* expression before and after drought stress, heat stress, and co-drought and heat stress. D_1 and D_6 represent 1 h and 6 h after drought stress treatment of wheat, respectively; H_1 and H_6 represent 1 h and 6 h after heat stress treatment of wheat, respectively; DH_1 and DH_6 represent 1 h and 6 h after co-drought and heat stress treatment of wheat, respectively; CK represents no stress treatment of wheat. The red, white and blue cells represent the highest, medium, and lowest gene expression levels, respectively. The colour scale represents Log_2_ expression values.

To investigate the tissue-specific expression of *TaATLa* genes, expression data from seven tissues (leaf, root, shoot, spike, stem, rachis, and grain) at six developmental stages (seedling stage, three-leaf stage, five-leaf stage, heading stage, anthesis stage, and grain-filling stage) were analyzed using the Wheat Expression Browser (http://www.wheat-expression.com/, accessed on 26 February 2024) and visualized using TBtools (v2.061) (Figure 5a, Table S4). The analysis revealed that *TaATLa5* exhibited the highest expression, particularly in the root and stem at various stages, while *TaATLa3* had the lowest. *TaATLa4* showed significant expression in the stem during the grain-filling stage, and *TaATLa2* was primarily expressed in the root. Most other *TaATLa* family members displayed low expression across all samples, suggesting diverse roles in wheat growth and development.

Next, we analyzed *TaATLa* gene expression under abiotic (drought, heat, and co-drought and heat) stress conditions and biotic (powdery mildew and stripe rust) using publicly available transcriptome data (Figure 5b, Figure S3). In response to drought stress, *TaATLa1* was markedly upregulated 6 h post-treatment, while *TaATLa3* was downregulated (Figure 5b). Heat stress induced the upregulation of *TaATLa5-7B*, *-7D*, and *TaATLa6*, and *TaATLa4* were downregulated 1 h after heat exposure but upregulated at 6 h. Under combined drought and heat stress, all *TaATLa* genes, except *TaATLa2*, were downregulated after 1 h. After powdery mildew exposure, *TaATLa6* was upregulated at 24 h while *TaATLa2* and *TaATLa4* responded at 48 h (Figure S3). *TaATLa1*, *TaATLa5*, and *TaATLa6* were upregulated following stripe rust stress at 24 h. *TaATLa6* was particularly responsive to both abiotic and biotic stresses. These results indicate that *TaATLa* genes likely play an important role in stress tolerance.

### 2.8. Heterologous Expression of TaATLa Genes in Yeast

**Figure 6:**
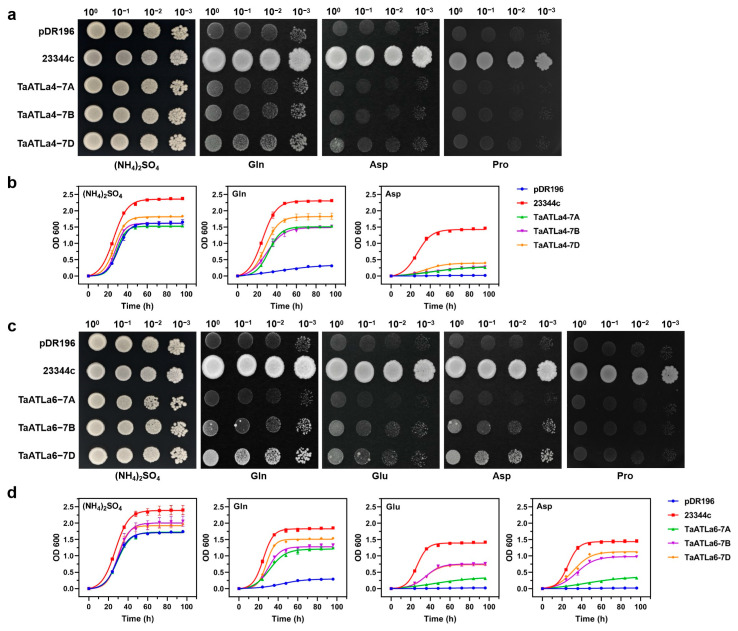
Yeast 22Δ10α growth complementation assay with an amino acid as the sole nitrogen source. (**a**) Images of yeast mutants transformed with *TaATLa4-7A*, *-7B*, *-7D* or empty vector pDR196 growth on YNB solid media were taken after 72 h at 28 °C. The 23344c (wild-type yeast strain) served as positive control. (**b**) Growth rates of yeast mutants transformed with *TaATLa4-7A*, *-7B*, *-7D* or empty vector pDR196. OD (Ab600) were measured at 24 h, 36 h, 48 h, 60 h, 72 h, 84 h, and 96 h (n = 3). (**c**) Images of yeast mutants transformed with *TaATLa6-7A*, *-7B*, *-7D* or empty vector pDR196 growth on YNB solid media were taken after 72 h at 28 °C. (**d**) Growth rates of yeast mutants transformed with *TaATLa6-7A*, *-7B*, *-7D* or empty vector pDR196.

To determine whether TaATLas are involved in amino acid transport, we used a mutant yeast strain, 22Δ10α, which is deficient in 10 amino acid transporter genes and unable to thrive in media containing amino acids other than arginine. The results confirmed that *TaATLa1* could facilitate yeast growth in a medium supplemented with 3 mM glutamate (Glu), Asp, or glutamine (Gln) [32]. Additionally, TaATLa4 and TaATLa6 exhibited similar amino acid uptake functions, promoting growth in the medium with Gln, Glu, or Asp (Figure 6a,c). In contrast, *TaATL2s*, *TaATL3s*, and *TaATL5s* did not affect growth compared to the negative control (empty vector pDR196).

To evaluate the transport efficiency of TaATLa4s and TaATLa6s, we examined the yeast growth rate in YNB liquid media with Glu, Asp, or Gln as the sole nitrogen source. The results indicated that although wild-type strain 23344c exhibited the highest growth rate, yeast cells expressing *TaATLa4s* or *TaATLa6s* demonstrated superior growth rates compared to the negative control, with TaATLa4-7D and TaATLa6-7D showing the strongest ability to utilize amino acids compared to their A and B orthologues (Figure 6b,d). Moreover, all transformants displayed a more pronounced capacity to utilize Gln over Glu and Asp.

### 2.9. Heat Tolerance Function Analysis of TaATLa4s and TaATLa6s in Yeast Heterologous Expression System

**Figure 7:**
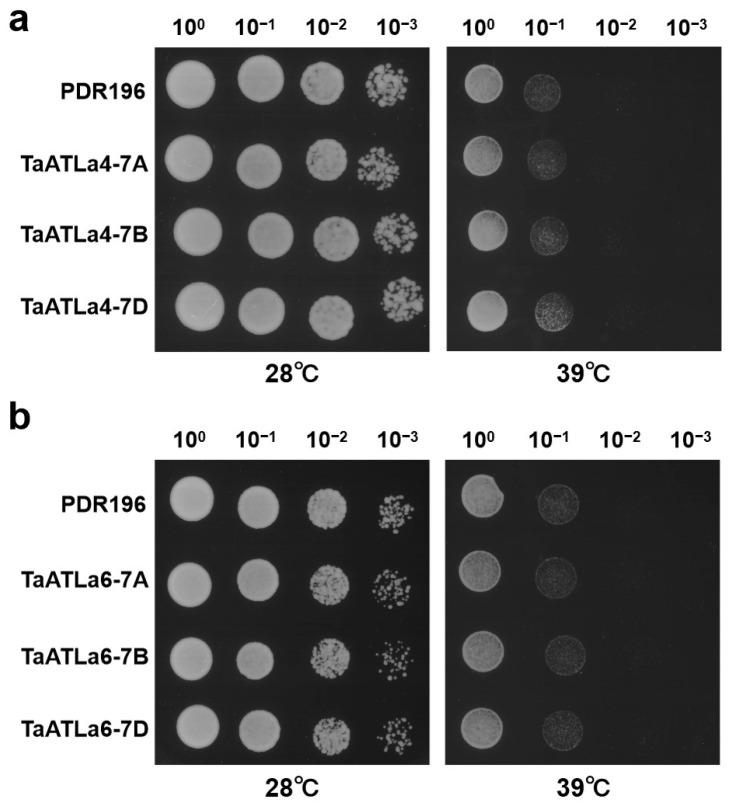
Expression of *TaATLa4* and *TaATLa6* in yeast to determine their responses under high temperature stress. (**a**) Images of yeast mutant 22Δ10α transformed with *TaATLa4-7A*, *-7B*, *-7D* or empty vector pDR196 growth on synthetic defined media lacking uracil (SD-Ura) solid medium after 72 h at 28 °C and 39 °C, respectively. (**b**) Images of yeast mutant 22Δ10α transformed with *TaATLa6-7A*, *-7B*, *-7D* or empty vector pDR196 growth on SD-Ura solid medium after 72 h at 28 °C and 39 °C, respectively.

Transcriptome analysis revealed that heat treatment for 6 h upregulated the expression of *TaATLa4* and *TaATLa6*. To further investigate their potential role in heat tolerance, a yeast heterologous expression system was employed and exposed to heat stress (Figure 7). The results demonstrated that, in contrast to the control yeast strains, which exhibited normal growth at 28 °C, all transformed yeast strains exhibited slow growth to varying extents under heat stress conditions (39 °C). Notably, the 22Δ10α strain transformed with *TaATLa4-7D* displayed enhanced tolerance to heat stress (Figure 7a,b), suggesting that *TaATLa4-7D* may have a positive regulatory role in heat tolerance.

## 3. Discussion

AATs play a vital role in plant development and response to environmental stresses, such as abiotic and pathogenic factors [18]. As a substantial subfamily of the AAT family, ATLa proteins have been identified in numerous species, such as *Phaseolus vulgaris* [2], rice [4], foxtail millet [7], and wheat [9]. This study provided a comprehensive analysis of *TaATLa* genes in wheat. These analyses covered their physicochemical properties, structural features, and expression patterns, and validated their amino acid transport functions through yeast heterologous expression.

In this research, we identified 18 *TaATLa* genes distributed uniformly across the A, B, and D subgenomes of wheat (Figure 1a and Figure 3a), suggesting no significant variation in abundance at the subgenome level. The number of *ATLa* genes in wheat is approximately 3.60, 2.57, and 2.25 times that in *Arabidopsis* (diploid), rice (diploid), and potato (tetraploid), respectively [12]. This increase is likely linked to allohexaploidy, which promotes gene proliferation [7]. Gene expansion typically occurs through two principal mechanisms: tandem duplication and segmental duplication [25]. Tandem duplication typically results in genes being closely clustered on the same chromosome, while segmental duplication leads to extensive gene amplification across the genome [33,34,35]. Interestingly, a genome-wide survey of the *AAT* gene family in wheat showed no evidence of tandem duplication among the *TaATLa* genes [12], suggesting that their expansion is primarily driven by segmental duplication.

Collinearity analysis across species showed that only three homologous *TaATLa1* genes on Chromosome 3 in wheat have six covariates with *Arabidopsis* (Figure 2), suggesting that *TaATLa1-3A*, *-3B*, and *-3D* are foundational genes in the subfamily’s evolutionary process. It can be speculated that homologous pairs between *Triticum aestivum* and *Arabidopsis* were established before the divergence of monocotyledons and dicotyledons, a hypothesis supported by previous research [24].

Gene duplication events often result in the preservation of primary functions and expression patterns of genes [36]. The comparable expression patterns of most *TaATLa* homologous genes across different subgenomes during developmental stages and under stress conditions (Figure 5) suggest a potential overlapping function among *TaATLa* genes. However, previous research has demonstrated significant functional and expression divergence among duplicated genes due to strong selection pressures [35]. Consequently, certain duplicated *TaATLa* genes may exhibit distinct expression patterns and responses to biotic and abiotic stresses. For example, *TaATLa5* exhibited the highest expression at various stages, while *TaATLa3* displayed the lowest (Figure 5a). Under drought stress, *TaATLa1* was upregulated, contrasting with the downregulation of *TaATLa3* (Figure 5b). In functional assays, TaATLa4 specifically transported Gln and Asp, whereas TaATLa6 transported Gln, Glu, and Asp (Figure 6). This mirrors findings in the tea plant (*Camellia sinensis* L.), where the high-affinity amino acid transporter CsLHT1 exhibited greater substrate specificity than the low-affinity transporter CsLHT6 [37]. These findings indicate that chromosomal duplication not only increased the number of *TaATLa* genes but also provided the genetic material necessary for their functional divergence, ultimately contributing to the evolution of new structural and functional roles. Consequently, further detailed functional analysis of each *TaATLa* gene is essential to fully understand their specific roles.

MiRNAs are small non-coding RNA molecules, typically 21–22 nucleotides long, that play crucial roles in regulating essential biological processes in plants and animals. The interaction between miRNAs and *TaATLa* genes provides an avenue for the precise genetic engineering of *TaATLas* through miRNA mediation. MiRNAs are known not only for their role in regulating plant growth but also for managing phenotypic plasticity in response to environmental stimuli, such as temperature, light, and nutrients [38]. The involvement of miR167 family members, such as tae-miR167a, tae-miR167b, and tae-miR167c-5p, in targeting *TaATLa2-6A*, *-6B*, and *-6D* suggests their potential role in regulating plant propagation and root development. The miR167 family has been associated with the biosynthesis of phytohormones that affect fertility and adventitious rooting in plants [39,40]. Similarly, tae-miR9657a has been identified as targeting a nucleosome/chromatin assembly factor, which is critical for DNA replication during cellular proliferation [41]. Therefore, *TaATLa3-6D*, predicted to be targeted by tae-miR9657a, may play a role in promoting cell proliferation, which is crucial and beneficial for plant development. Additionally, various miRNAs, such as tae-miR9677b, tae-miR167, and tae-miR5384-3p, were found to target homologous *TaATLa* genes, indicating their potential participation in early meiosis in wheat. Given the intricate nature of the wheat genome, which has not been fully sequenced [12], research on miRNAs in wheat remains limited. This underlines the need for further exploration and validation of miRNA-*TaATLa* gene regulatory networks to uncover their functional roles in wheat development and stress responses.

Amino acid transport in plants is significantly influenced by environmental signals, such as heat, drought, and high salinity [42]. In rice, the genes *OsATL6* and *OsAAP11* from the *OsAAT* family were significantly upregulated in response to drought stress [4], while, in wheat, *TaAAP3* and *TaATLb13* responded to both drought and high temperatures [12]. Similarly, this study showed that *TaATLa3* was notably downregulated, while *TaATLa1* was upregulated under drought stress. In contrast, *TaATLa4* and *TaATLa6* were upregulated in response to heat stress (Figure 5b). These stress-related expression patterns may be linked to cis-acting elements in the promoters of these genes [43,44]. For instance, *TaATLa1*, *TaATLa2*, and *TaATLa6* contain MBS cis-elements, which are involved in drought induction (Table S3). Additionally, abscisic acid (ABA) and salicylic acid (SA) are key regulators of plant responses to high-temperature stress. When exposed to high temperatures, plants rapidly accumulate ABA, which activates downstream heat-resistant genes to enhance stress resistance [45]. SA primarily improves plant heat tolerance by activating antioxidant genes, helping to eliminate reactive oxygen species [46]. The promoter region of *TaATLa* genes contains ABRE cis-acting elements responsive to ABA and TCA-elements responsive to SA, which may enhance wheat’s tolerance by regulating gene expression at the promoter level (Table S3). Future studies should focus on further experimental verification of the cis-acting elements and the predicted regulatory networks of interest, specifically regarding their roles in gene regulation under various conditions.

This study conducted a functional analysis of *TaATLa4* and *TaATLa6*, genes highly expressed in wheat under high-temperature stress, using a yeast heterologous expression system to investigate their roles in heat tolerance (Figure 7). *TaATLa4-7D* expression in yeast enhanced the heat tolerance of the complementary strain, indicating its potential role in regulating amino acid transport under heat stress. In plants, proline biosynthesis occurs through two distinct pathways: the glutamate pathway and the ornithine pathway [47]. As *TaATLa4-7D* overexpression increased glutamine absorption, a precursor to proline synthesis in yeast, it is hypothesized that glutamine accumulation in cells may increase proline levels, thereby improving their ability to withstand heat stress. This suggests that *TaATLa4* may play a crucial role in enhancing heat tolerance by modulating proline biosynthesis. Although these findings lay an important foundation for understanding the function of *TaATLa* genes in abiotic stress responses, further investigation is needed to verify whether the heat tolerance conferred by *TaATLa* subfamily genes in yeast also applies to wheat.

Nitrogen availability is critical for optimal plant growth and development, significantly influencing crop yield and grain quality [20,48]. The challenges of global climate change are becoming increasingly serious, wheat yield and quality are especially vulnerable to abiotic stresses [43]. The yeast heterologous complementation assay demonstrated that TaATLas, particularly TaATLa4 and TaATLa6, transport amino acids such as Gln, Glu, and Asp (Figure 6a,c). These amino acids function as signaling molecules that regulate transcription factors under nitrogen stress, as well as precursors for other nitrogen-containing compounds essential for wheat grain developing [32,49,50]. Considering the function of TaATLa1 [32] in amino acid transport and NUE, it can be inferred that TaATLa4 and TaATLa6 also contribute to improving NUE by modulating nitrogen assimilation pathways and responses to abiotic stress. Future studies should prioritize the functional exploration of the TaATLa subfamily, particularly through field trials utilizing wheat lines edited for targeted *TaATLa* genes. This will enable a more thorough understanding of their potential to enhance crop performance and stress resilience, which is crucial for advancing wheat breeding programs to address environmental climate changes and maintain yield and quality.

## 4. Materials and Methods

### 4.1. Identification of ATLa Subfamily Genes in Wheat

Genomic data for wheat, rice, and *Arabidopsis* were retrieved from the Ensembl Plants Database (https://plants.ensembl.org/index.html, accessed on 7 February 2024). The IDs of five *AtSN1L* and six *OsATL* genes were obtained from the existing literature on *ATL* gene families. Utilizing TBtools (v2.061) and these gene IDs, the protein sequences of ATL from *Arabidopsis* and rice were extracted. A BLAST search was conducted within the wheat database on Ensembl Plants, filtering candidate TaATLa subfamily proteins based on an E-value ≤ 10^−10^ and homology exceeding 70%. All candidate proteins were subsequently analyzed using the Pfam database (https://ngdc.cncb.ac.cn/databasecommons/database/id/186, accessed on 7 February 2024) and the NCBI Batch Web CD-Search Tool (https://www.ncbi.nlm.nih.gov/Structure/bwrpsb/bwrpsb.cgi, accessed on 7 February 2024). Orthologues of the *TaATLa* subfamily genes in *Triticum dicoccoides*, *Triticum urartu*, *Aegilops tauschii*, and *Triticum turgidum* were identified through the Ensembl Plants Database and designated based on their chromosome positions. Comprehensive details regarding the *TaATLa* subfamily genes, including gene structure, open reading frame (ORF) length, and TM regions, were retrieved from the Ensembl Plants Database. MW and PI were determined using ExPAsy (https://web.expasy.org/protparam/, accessed on 7 February 2024). TBtools (v2.061) was employed to extract amino acid length information from GFF3 files, while subcellular localization of TaATLa proteins was predicted using Plant-mPLoc (http://www.csbio.sjtu.edu.cn/bioinf/plant-multi/, accessed on 8 February 2024).

### 4.2. Phylogenetic Analysis

To investigate evolutionary relationships, the full-length ATL proteins from wheat, *Arabidopsis*, rice, *Triticum dicoccoides*, *Triticum urartu*, *Aegilops tauschii*, and *Triticum turgidum* were aligned using the MUSCLE program in MEGA11 with default settings. Subsequently, a phylogenetic tree was constructed utilizing the neighbor-joining method, a distance-based algorithm for inferring evolutionary relationships. A bootstrap value of 1000 was specified to assess the reliability of the tree topology. All other parameters remained at their default settings. The resulting evolutionary tree was visually enhanced using the iTOL (v6.9) web application (https://itol.embl.de/, accessed on 17 February 2024).

### 4.3. Chromosomal Positioning and Gene Duplication Events Analysis

The chromosomal locations of *TaATLa* genes were determined based on the annotation data of the wheat genome. This information was then analyzed using TBtools (version 2.061) for Gene Location Visualization, allowing for a clear display of the distribution of these genes across the wheat chromosomes. Circos plots were also generated to visually represent gene positioning. To detect gene replication events, Multiple Collinear Scanning Toolkits (MCScanX) in TBtools (version 2.061) were employed, encompassing species such as wheat, *Arabidopsis*, rice, *Aegilops tauschii*, and *Triticum dicoccoides*.

### 4.4. Calculation of Ka/Ks Values

In genetics, the Ka/Ks ratio, also known as the non-synonymous to synonymous substitution ratio, indicates the relationship between non-synonymous substitution (Ka) and synonymous substitution (Ks) in protein-coding genes. This ratio helps to ascertain whether selection pressure is influencing protein-coding genes. In this study, the Ka/Ks ratio was calculated using TBtools (v2.061) software.

### 4.5. Analysis of Gene Structure and Motifs for TaATLas

The exon and intron structural information for the *TaATLa* subfamily members was obtained from the wheat GFF3 file, and the gene structure was analyzed using TBtools (v2.061) Gene Structure View. The conserved protein motifs of the TaATLa proteins were identified using the MEME Suite 5.5.5 online analysis platform (https://meme-suite.org/meme/tools/meme, accessed on 18 February 2024), allowing for the detection of up to 10 conserved motifs.

### 4.6. Cis-Element Analysis in the Promoter

The upstream promoter region (2000 bp) of the *TaATLa* gene subfamily was retrieved from the Ensembl Plants Database. Cis-elements within these promoters were analyzed utilizing the PlantCARE database (https://bioinformatics.psb.ugent.be/webtools/plantcare/html/, accessed on 14 February 2024).

### 4.7. Forecasting MiRNA Target Locations for TaATLa Subfamily Genes

The mRNA sequences of the *TaATLa* subfamily genes were obtained from the Ensembl Plants Database and submitted to the pSNaTarget online platform (https://www.zhaolab.org/psRNATarget/, accessed on 23 February 2024) to predict potential miRNA target sites within the *TaATLa* genes. Predicted miRNA-target interactions were visualized using Cytoscape software (version 3.10).

### 4.8. Expression Pattern Analysis

We downloaded RNA sequence data from seven tissues (leaf, root, shoot, spike, stem, rachis, and grain) across six stages (seedling, three-leaf, five-leaf, heading, anthesis, and grain-filling) during the entire wheat growth period and under five stress conditions (drought, heat, co-drought and heat stress, powdery mildew, and stripe rust) to analyze the expression profile of *TaATLas* from the Wheat Expression Browser (http://www.wheat-expression.com/, accessed on 26 February 2024). The raw data were processed with log_2_ (tpm + 1) transformation and visualized with the Heat Map functionality in Tbtools (v2.061).

### 4.9. Heterologous Expression of TaATLa Subfamily Genes in Yeast

The full-length coding sequences of *TaATLa* genes were amplified using KOD FX DNA Polymerase (Toyobo, Osaka, Japan) and cloned into the yeast expression vector pDR196 at the Spe I and EcoR I restriction sites [51]. The primers used for cloning are listed in Table S5. Complementation analysis of *TaATLa* genes in yeast was performed using the 22Δ10α mutant strain described by Besnard et al. [52] and the wild-type strain 23344c as a positive control, which cannot utilize lysine, histidine, or cysteine as the sole nitrogen source. The specific experimental methods employed in this study were based on Chen et al.’s previous work on the heterologous expression of *TaATLa1* in yeast [32]. Data were analyzed and presented using GraphPad Prism 8.0.1.

### 4.10. Characterization of Heat Tolerance of Yeast Strains Carrying TaATLa4 and TaATLa6

The yeast expression vectors pDR196 harboring the target genes *TaATLa4-7A*, *-7B*, *-7D*, and *TaATLa6-7A*, *-7B*, *-7D* were individually transformed into the mutant yeast strain 22Δ10α using the lithium acetate/single-stranded carrier DNA/polyethylene glycol (LiAc/SS-DNA/PEG) method [53]. The transformants were selected on solid agar containing 8 g/L Ura Minus Media (FunGenome, Beijing, China) and 20 g/L glucose. Colonies were incubated in synthetic defined media lacking uracil (SD-Ura) liquid medium at 28 °C until the optical density (OD) reached 1.0. Yeast cells were then harvested by centrifugation at 2500× *g* for 3 min, washed twice with sterilized water, and serially diluted to 10^−1^, 10^−2^, and 10^−3^. Droplets (5 μL) of each dilution were spotted onto SD-Ura solid medium and incubated at 28 °C and 39 °C for 3 d.

## 5. Conclusions

This research conducted a comprehensive genome-wide identification and functional analysis of the *TaATLa* subfamily genes. Evolutionary analysis revealed that these genes have undergone continuous duplication and functional diversification, enhancing our understanding of complex traits and evolutionary processes. Members of orthologous gene groups may exhibit similar functions based on their gene structures and conserved domains. The heterologous expression of *TaATLa* genes in yeast demonstrated that TaATLa4s and TaATLa6s possess amino acid transport functions, probably contributing to increased grain protein content by transporting Gln, Glu, or Asp. Furthermore, *TaATLa* genes exhibited diverse expression patterns in different tissues under biotic and abiotic stresses, as well as in response to plant hormones. Given that *TaATLa4-7D* promoted heat tolerance in the complementary yeast strain, it is hypothesized that its overexpression may have a similar function in wheat, which requires further confirmation. With the advancements in biotechnology and functional genomics, such studies are expected to identify key genes sensitive to abiotic stress and elucidate the genetic improvement of *ATL* genes in major food crops by selecting appropriate genotypes to adapt to environmental and climate changes.

## Figures and Tables

**Table 1:** Genomic information and characterization of proteins encoded by *ATLa* gene subfamily members in wheat.

No.	Gene Name	Gene ID	Gene Structure		ORF (bp)	Protein			TM Region	Subcellular Localization
			Length (bp)	Intron		Size (aa)	MW (kD)	PI		
1	*TaATLa1-3A*	TraesCS3A02G346700.1	1994	4	1380	459	48.137	9.27	10	Plasma Membrane
2	*TaATLa1-3B*	TraesCS3B02G378500.1	1994	4	1377	458	47.996	9.27	10	Plasma Membrane
3	*TaATLa1-3D*	TraesCS3D02G340400.2	1982	4	1380	459	47.991	8.64	10	Plasma Membrane
4	*TaATLa2-6A*	TraesCS6A02G170700.1	2293	4	1377	458	50.175	6.18	10	Plasma Membrane
5	*TaATLa2-6B*	TraesCS6B02G198900.1	2127	4	1377	458	50.205	6.31	10	Plasma Membrane
6	*TaATLa2-6D*	TraesCS6D02G160400.1	2116	4	1377	458	50.243	6.31	10	Plasma Membrane
7	*TaATLa3-6A*	TraesCS6A02G288200.1	7247	4	1347	448	47.896	7.05	10	Plasma Membrane
8	*TaATLa3-6B*	TraesCS6B02G317700.1	6498	4	1338	445	47.652	7.51	10	Plasma Membrane
9	*TaATLa3-6D*	TraesCS6D02G270800.1	4757	4	1338	445	47.521	7.51	10	Plasma Membrane
10	*TaATLa4-7A*	TraesCS7A02G212200.1	4584	4	1386	461	49.776	6.82	12	Plasma Membrane
11	*TaATLa4-7B*	TraesCS7B02G119000.1	3784	4	1386	461	49.743	6.49	12	Plasma Membrane
12	*TaATLa4-7D*	TraesCS7D02G213900.1	3503	4	1386	461	49.763	6.92	12	Plasma Membrane
13	*TaATLa5-7A*	TraesCS7A02G391600.1	2178	4	1377	458	49.757	6.58	10	Plasma Membrane
14	*TaATLa5-7B*	TraesCS7B02G293500.1	2177	4	1377	458	49.796	6.58	10	Plasma Membrane
15	*TaATLa5-7D*	TraesCS7D02G387200.1	2159	4	1377	458	49.766	6.58	10	Plasma Membrane
16	*TaATLa6-7A*	TraesCS7A02G517100.1	1461	0	1461	486	51.675	5.92	11	Plasma Membrane
17	*TaATLa6-7B*	TraesCS7B02G433400.1	1476	0	1476	491	52.199	6.49	11	Plasma Membrane
18	*TaATLa6-7D*	TraesCS7D02G507300.1	1476	0	1476	491	52.141	6.34	11	Plasma Membrane

## Data Availability

Data are contained within the article and Supplementary Materials.

## Supplementary Materials

The following supporting information can be downloaded at: https://www.mdpi.com/article/10.3390/ijms252212454/s1.
