# Supplementary material for: Bioinformatics and Expression Analyses of the TaATLa Gene Subfamily in Wheat (Triticum aestivum L.)
Source: Int J Mol Sci. 2024 Nov 20;25(22):12454. doi: 10.3390/ijms252212454 (PMC11594669; doi:10.3390/ijms252212454)
Supplement: Supplementary file 1 [file ijms-25-12454-s001.zip › Supplementary figures S1-S3.pdf]

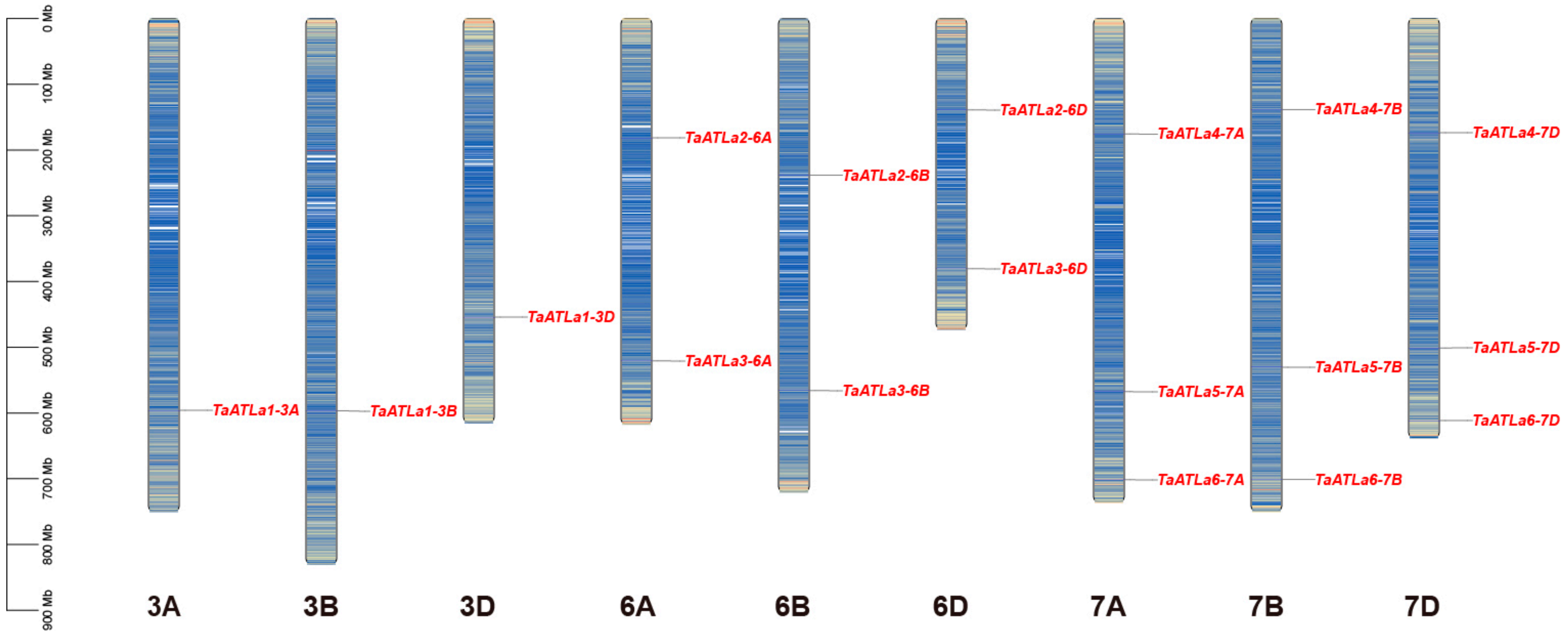

Figure S1. Chromosomal distributions of *ATLa* gene subfamily members in wheat. The basic unit indicates a chromosome length of 100 Mb. The interior of the chromosome is filled with gene density, with colours ranging from blue to red indicating a gradual increase in gene density.

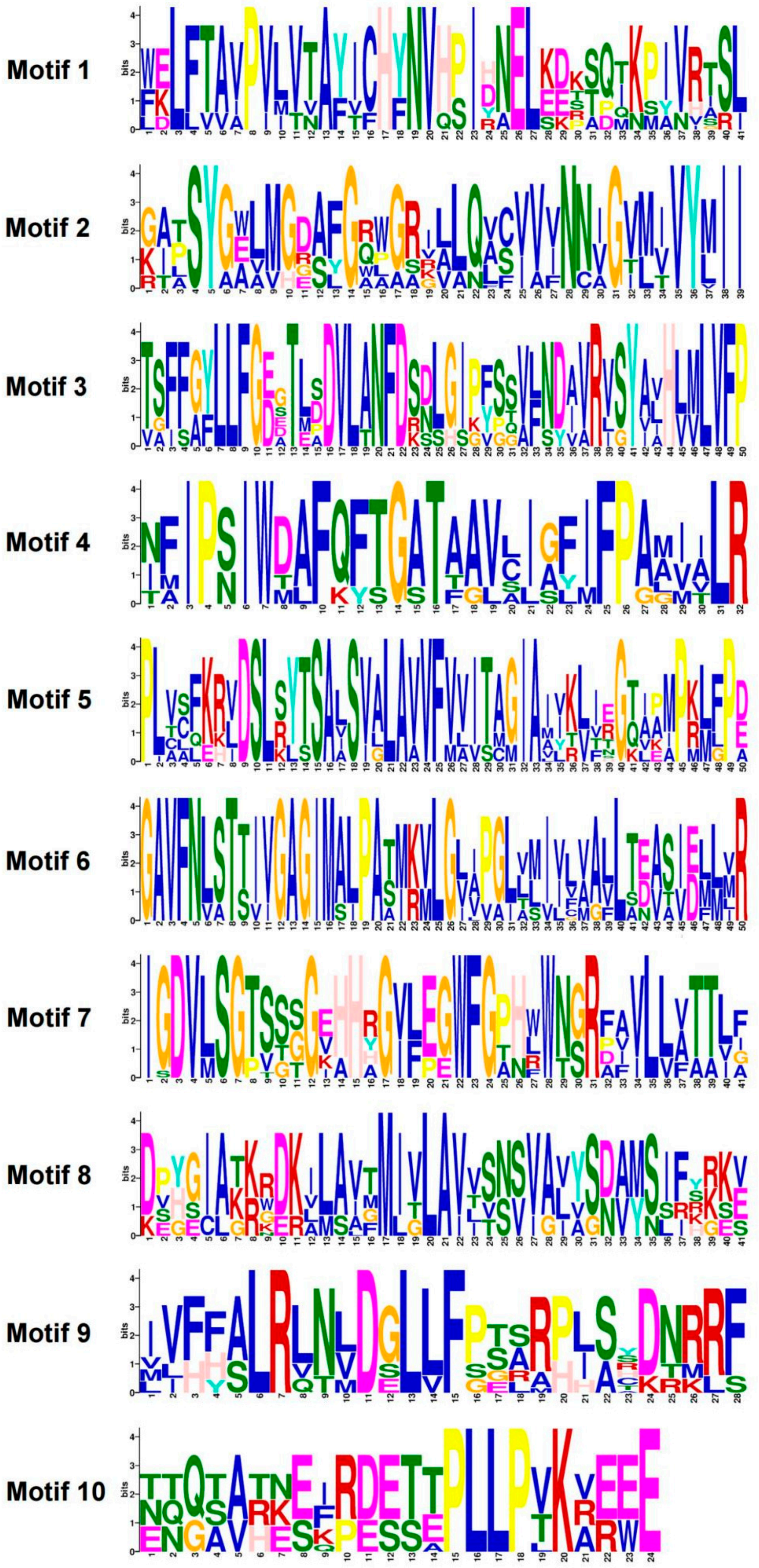

Figure S2. Motif logos generated by the MEME online website.

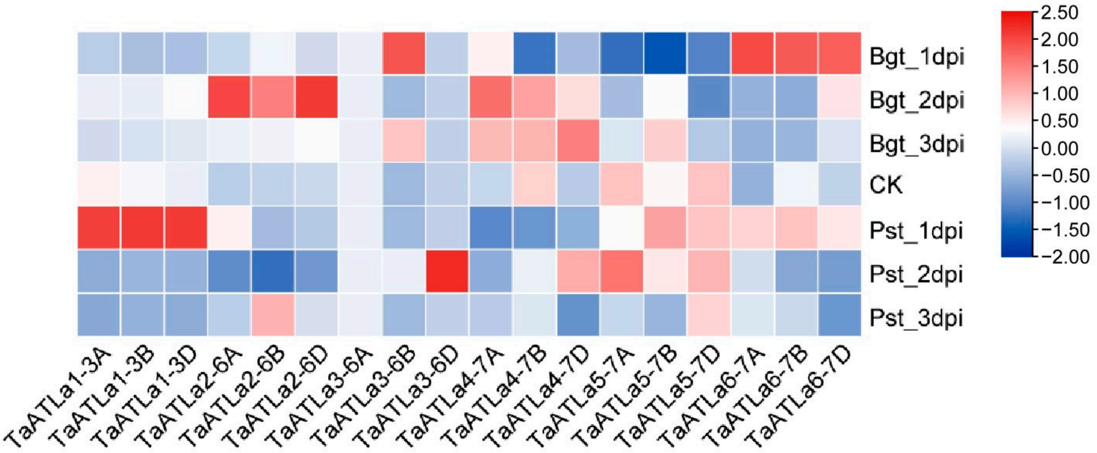

Figure S3. Expression profiles of *TaATLas* before and after the injection of powdery mildew and stripe rust. Bgt_1dpi, Bgt_2dpi, and Bgt_3dpi represent 24 h, 48 h, and 72 h after powdery mildew pathogen was injected into wheat leaves; Pst_1dpi, Pst_2dpi, and Pst_3dpi represent 24 h, 48 h, and 72 h after stripe rust pathogen was injected into wheat leaves; CK represents the control leaves not injected with powdery mildew or stripe rust. The red, white and blue cells represent the highest, medium and lowest gene expression levels, respectively. The colour scale represents Log_2 expression values.
